# The Diagnosis and Surgical Treatment of Nephrolithiasis from the Viewpoint of the General Practitioner

**Published:** 1902-05

**Authors:** Ramon Guitéras

**Affiliations:** New York


### BUFFALO MEDICAL JOURNAL.

VOL. XLI.—LVII. MAY, 1902. No. 10.

### ORIGINAL COMMUNICATIONS.

### The Diagnosis and Surgical Treatment of Nephrolithiasis From the Viewpoint of the General Practitioner.

WITH NOTES OF ILLUSTRATIVE CASES.[1]

By RAMON GUITÉRAS, M.D., New York.

ONE of the subjects classified as lying on the border-line between the two vast domains—medicine and surgery—and that therefore brings into close community the work of the physician and surgeon, is stone in the kidney.

*Causes and Pathology*.—The etiology of nephrolithiasis will not be discussed in detail in this paper, as it involves many an abstruse question in the domain of pathologic metabolism, to encroach upon which lies beyond the intention of the writer.

It must be said, however, that there are certain predisposing factors that have been noticed in connection with the occurrence of stone, preëminent among which is that of age; for it has been conclusively proved that this disease is most often met with in persons in the middle of life's highway,—that is, between the ages of 20 and 50 years. Women are more frequently affected than men.

Country and race have some influence upon the development of stone, as warm countries seem to favor it. For example, renal calculi are very common in India. Renal stones are generally stated to be more frequent among the Anglo-Saxons than among the Latin races, especially the Spanish and Spanish Americans. I think, however, that such conclusions are due to the fact that better statistics and more complete reports on this subject have been published in Anglo-Saxon literature than elsewhere. Personally, I have found stone more common in this country among the Italians than in any other race.

1. An address read before the Buffalo Academy of Medicine, January 7, 1902.

Sedentary life with luxurious habits and so-called "high-living" without proper exercise and sufficient oxidation also lead to its formation.

There are two varieties of stone, primary and secondary. The primary stones are formed by the precipitation of substances formed in the kidney without any previous pathological changes in the parenchyma, the stroma, the pelvis or the calyces. Secondary stones are the results of pathological processes in these localities and are formed when certain salts are precipitated, owing to the decomposition of the urine. Of course, a calculous may be originally primary, and by its presence so irritate the surrounding tissues that lesions develop which give rise to secondary calculous deposits over the primary calculus.

Of the primary variety of calculi we have those formed in acid urine and those formed in alkaline urine. The varieties developing in acid urine consist of one or more of the following substances: uric acid, urates, calcium oxalate, cystin, and xanthin. Those formed from alkaline urine consist of calcium carbonate, acid phosphates or calcium, or basic phosphate of calcium.

Secondary calculi are precipitated from alkaline urine rendered so by ammoniacal decomposition, due to some local infection or inflammation. They consist of phosphate of ammonium, phosphate of magnesium, or of phosphate of ammonium and magnesium.

Oxalate of calcium calculi occur twice as frequently as either the uric acid or the phosphatic variety, and produce the worst cases of nephrolithiasis. Of the different varieties enumerated the most frequent in their relative order are: oxalate of lime, uric acid and phosphatic compounds.

Calculi vary greatly in number from one to over 200, and in size from grains of sand to masses, weighing 5 pounds. The usual weight of a calculous is from ½ an ounce to 2 ounces.

Stones are more often found in the right kidney than in the left, while the frequency of stones occurring in both kidneys is variously estimated at from 5 to 50 per cent.

The effects of renal calculi must be considered in connection with the causes and pathology, as a knowledge of the secondary changes in the kidney, the pelvis and the ureter, as well as in other parts affected by renal stones is of importance to the surgeon. The effects of primary calculi may be conveniently grouped under two headings, following the classification of Albarran,[1] Morris,[2] and other writers on the subject, as the aseptic changes, and the secondary or septic changes.

The aseptic changes of primary calculi begin with a secondary nephritis of calculous origin (Nephrite diathésique of Albarran). This is a nephritic inflammation, beginning in the renal epithelium, later involving the interstitial framework of the organ, and according to Albarran, is always present in calculous kidneys. When the calculi have reached a considerable size and have remained in the kidney for a long time, especially if they cause obstruction of the ureter, other lesions may become associated with this nephritis. The first of these is atrophy, which is the result of the interstitial nephritis; in other cases the mechanical obstruction produced by the stone gives rise to a hydronephrosis, and finally sometimes the perirenal fat hypertrophies to such an extent that it forms a large lipomatous tumor within which the atrophied and shrunken kidney is enclosed.

The septic lesions of primary calculi are secondary to the aseptic changes. The source of the sepsis is usually an ascending infection from the urethra and bladder (Tuffier) or the bacteria may reach the kidney through the blood or the lymph. The predisposing factors to septic infection which exist in calculous kidney are the traumatic effects of the stones, the calculous nephritis, the urinary retention which they cause, and the capillary congestion of the organ. The results of infection in calculous kidneys are pyelonephritis, pyonephrosis and perinephritis.

The pyelonephritis of renal calculi is characterised by a septic diffuse sclerous nephritis, which was described by Albarran. It has been noted, however, that when the sclerotic processes (which precede the pyelonephritis as results of the secondary aseptic nephritis, of which we have spoken), are sufficiently far advanced, the kidney itself seems better able to resist the invasion of bacteria, and this, according to Albarran, is the reason why nephrolithotomy has been successful in some cases in which there was a pyelitis.

The kidney affected with pyonephrosis as the result of infection and of calculous obstruction of the ureter, usually reaches a considerable size, and often presents a series of loculi filled with purulent material, and containing calculi which are difficult to dislodge from their cavities on account of adhesions. The

1. J. Albarran, Maladies du Rein et de l'Uretère, in Delbet et Le Dentu, Traité de Chirurgie, J. VIII. Paris, Baillère, 1899.

2. Henry Morris, Surgical Diseases of the Kidney and Ureter, Vol. II. London, Cassell, 1901.

abscess cavity may burst and the contents burrow in various directions.

The perirenal fatty capsule is often thickened and the seat of fibrous changes in the presence of renal calculi and infection. If the latter be virulent, there arises a true perinephritis and adhesions may bind the kidney to the surrounding organs,—a fact of importance to be remembered when operating in such cases, as in some instances adhesions exist between the kidney and vena cava. The perinephritis may, of course, suppurate; the pus may burrow in various directions, and may empty through fistulous openings.

The opposite kidney is often the seat of compensatory hypertrophy, in order to make up for the loss of renal tissue occasioned by the presence of stones, and for the atrophic changes in the renal parenchyma of the calculous organ. In other cases the opposite kidney may be the seat of secondary nephritis, due to the excessive strain, and to the great amount of uric acid, oxalates, and phosphates and the like (as the case may be) that are eliminated through it, and, finally, it may be the seat of the same forms of calculi as on the opposite side.

Suppression of urine results when the pelvic orifices of the ureters are occluded by stones. One kidney may thus be incapacitated, and then the other; or both may be put out of work altogether. If the stones become impacted in the ureter further down on their way to the bladder, they may cause ulceration, ureteritis or periureteritis, with the formation of fibrous tissue occluding the canal; or they may perforate into the retroperitoneal or peritoneal cavities, or into the intestine.

*Diagnosis.*—In considering the clinical features of nephrolithiasis we must take into account, first, that although most patients come to us with symptoms, there is a considerable proportion of cases in which the presence of stones remains unaccompanied by clinical signs. In such cases the stone either remains in the kidney in such a way that it cannot do damage, or is so small that it passes through the ureter into the bladder and is passed out in the urine, or else remains in the bladder as a nucleus for a vesical calculus. Bruce Clark found that in 13 of 24 autopsies on persons in whose kidneys calculi were present, there had been no symptoms whatever referable to nephrolithiasis. The fact is, the symptoms are not always in proportion to the extent of the disease, and I have often been surprised to find how few symptoms a patient will have when his kidneys have been nearly destroyed by the morbid process.

A second clinical group of cases is one in which the existence of stone is not suspected, because the train of symptoms does not point to any affections of the kidneys, but rather to the trouble in the bladder, the uterus, the ovaries, or the testicles. In such cases the symptoms are reflex in origin, as will be seen from what follows.

In a third group of cases, noted particularly by Morris, there is great mental distress and a febrile movement, but no renal colic occurs.

In the majority of cases there is pain in the lumbar region corresponding to the affected side, or in that side of the abdomen, although there may be a general abdominal pain. This pain often runs down into the groin, or the testes on the affected side. It is of varying degrees of intensity, from a dull ache to the excruciating, sharp, cutting pain of renal colic. It may be continuous, but generally follows exercise, or jolting, although cases have been reported when it occurred at night (Jackson, *British Medical Journal*, 1890, p. 67.)

It is renal colic, however, that usually causes the patient to consult a physician in cases of stone in the kidney, especially if it be associated with or followed by hematuria. It occurs when a freely movable stone of a certain size begins to engage in the mouth of the ureter, or to descend along this canal. The forces which propel a stone along the ureter in such cases are said to be three-fold, viz.: the pressure of pent-up urine behind; the worm-like contraction of the ureter under the irritation produced by a foreign body, and the alternating positive and negative pressure of the act of vomiting which often accompanies the attack of colic.

The clinical picture of a patient in the throes of renal colic is not easily forgotten when once seen. The pallid, anxious face thrown into an expression of most excruciating suffering, and covered with cold sweat; the characteristic posture of the body with flexed trunk and thighs, restlessly moving from one position to another in the agony of the paroxysm—these are sufficiently typical of renal colic to direct our attention to the possibility of stone in the kidney.

The pain in renal colic is the central symptom of the syndrome. Its intensity is said to exceed that of any other pain known to mankind, but this is a difficult question to decide.

At all events, patients have been know to commit suicide in the course of an attack. The pain is acute, paroxysmal, has its chief seat in the loin or in the side of the abdomen, and radiates along the ureter toward the testicles or the labia majora, or occasionally into the thigh, or even into the opposite side of the abdomen. The testes may be retracted during the attack, and may afterward be swollen and tender.

A chill frequently precedes the access of pain, and during the attack the patient may faint or have convulsions. Nausea, vomiting and retching often complicate matters during the attack, and painful and frequent passages of urine tinged with blood, or of blood clots, commonly accompany the seizure. The paroxysm usually lasts from two to three hours, and, as a rule, terminates more or less abruptly, the patient feeling relieved and falling asleep from exhaustion.

In some cases the colicky pains are not so violent and represent a milder form of paroxysm, characterised by tenderness over the course of the ureter, and not accompanied by a sharply localised pain, but merely by a diffuse pain over the abdomen.

It is the cases in which the stone remains in the kidney or pelvis without giving rise to attacks of renal colic that present the greatest difficulties in diagnosis. These cases are characterised by the following cardinal symptoms: Pain, referable to the kidney, or some other organ in reflex relation to it, aggravated by motion, by jolts and jars and lessened by rest; hematuria and pyuria.

Reflex pains or referred pains are often met with in nephrolithiasis and frequently mislead the diagnostician. In such instances the renal region may be free from pain. The pain may be seated in the lumbar spines or may be felt in the sciatic nerve or the lumbar sensory nerves, as in lumbar sciatic neuritis. Guyon, who has studied the reflex pains of nephrolithiasis in detail, classifies them as follows: (1) The renorenal reflex. Pain in the opposite, supposedly healthy, kidney. Morris, in speaking of renorenal reflex says that the pain in the opposite kidney is of an aching character not spasmodic or colicky, is transient and occurs as an accompaniment of pain in the affected side. (2) The renovesical reflex, accompanied by pain in the vesical neck, and by frequent and painful micturition. (3) The renoureteral reflex. The pain is felt along the course of the ureter, which is found to be tender on pressure by rectum. (4) The renoövarian or renouterine reflex and their analogue, the renotesticular reflex are very misleading and not at all uncommon. (5) The gastrointestinal reflex consists of the phenomenon of "nervous dyspepsia," accounted for by the connection of the vagus with the renal plexus. In rare cases there are attacks of intestinal colic.

If pain be not the reason why a patient consults a physician for this disease it may be the presence of blood in the urine. This is one of the most frequent symptoms of renal calculi, and the bleeding occurs independently of the bloody urine which is passed during or following attacks of renal colic. Morris found it to be present in 41 out of 103 operated cases of stone. It must be said, however, that the amount of blood passed is usually scanty, and often only detectable on microscopic examination. It is aggravated by movement and by prolonged standing and lessened by rest in bed, and is due both to congestion and injury of the tissues by attrition on the part of the stone. Oxalic calculi being rougher than the others are more apt to cause hematuria. It consists of free blood, blood-casts, and frequently ureter-casts, consisting of elongated blood clots.

As the result of a pyelitis which usually occurs in the course of nephrolithiasis and may be slight, the urine contains minute quantities of pus, or the pus may be abundant, the urine having that offensive odor and cloudy precipitate which is characteristic of advanced pyelonephritis.

The examination of the urine of patients with renal stones is a subject of vast importance, which lies beyond the scope of this paper. It is always advisable to have urinary examinations performed by a pathologist or bacteriologist and not to trust to one's own examination, or to that of an interne, in doubtful cases.

The amount of urine is usually decreased. The reaction is, as a rule, markedly acid, except in cases of phosphatic calculi or advanced pyelitis; the specific gravity varies with the amount of dilutents taken; the color is usually darker than normal, and if hematuria be present blood is found mixed with the urine giving it a smoky-brownish or porter-like color. The urine often shows a sediment varying in color from whitish to dark-brown or red. The chemical examination usually shows the presence of albumin and of an excess of uric acid and urates, or of phosphates or of oxalates, as the case may be. When pyelitis is present especially, phosphates appear in excess and are deposited in the sediment.

Microscopic examination shows the presence of blood, pus, epithelium from the tubules, the renal pelvis, the ureter or the bladder; casts (hyaline, granular, epithelial, blood, pus or mixed), and bacteria, and often of small concretions of crystals or masses of crystalline sediments, such as those of calcium oxalate, triple phosphates, uric acid and urates, which point to the nature of the stone in the kidney.

When urine of this kind is found in a patient suffering from pains or colic in the region of the kidney, the pains coming usually during the day after exercise, and associated perhaps with frequency of urination, one may strongly suspect stone in the kidney on the side where the pain is felt, especially if there are no tubercle bacilli in the urine, or any signs of tuberculosis in other organs.

*Differentiation.*—The number of conditions with which stone in the kidney is confounded is very great, and to go into a detailed consideration of this phase of the question would extend this paper to the size of a monograph.

Stone in the kidney is more apt to be confounded with tuberculosis or tumor of that organ than with any other renal disease. The principal points of differentiation lie in the history. On the side of calculus there may be presence of gout, rheumatism, or lithemia in the individual or in the family, while in that of tuberculosis there may be a tuberculous history in the patient or his family. Tuberculosis of the kidney usually comes on more rapidly and the patient is more cachectic; the hematuria and the frequency of urination often take place at night as well as by day. In renal tuberculosis there may be an involvement of the genito-urinary tract elsewhere, as in the bladder, prostate or testes, and tubercle bacilli will sooner or later be found in the urine, while in cases of calculus the symptoms occur almost always during the day.

In cases of tumor of the kidney the malignancy of the growth is expressed by the cachexia and loss of weight which accompanies these cases. In such patients the hemorrhages are much more abundant and not necessarily associated with moving, or jolting. The organ is usually clearly outlined as a tumor; the urine may contain characteristic cells, and is not so apt to contain crystals. The pain is always in the back and loins.

A movable kidney may also give rise to a dull ache in this region or even to renal colic, but hematuria is always absent or slight, pyuria does not so often develop, and nephritis is not so commonly associated with movable kidney as with calculus. The former condition is generally found on the right side and most frequently in women. It is usually not accompanied by enlargement, and the kidney may be felt to be movable and may be replaced

Various diseases of the bladder, such as stone, tubercle, tumor, alone or associated with cystitis, congestion or cystitis due to stricture or prostatic hypertrophy, and gonorrheal cystitis, may give rise to pain in the abdomen or back. In these cases pyuria and hematuria may be present, but the epithelium in the urine will be found to be vesical and there will be no kidney elements present. A urethral and rectal examination associated with visual exploration of the bladder by means of an examining cystoscope, will clear up the diagnosis.

Other conditions likely to be mistaken for stone are a group of neuralgias, which may be either parietal, like sciatica or lumbago, intercostal, or inguinal; or else visceral, *i.e.*, renal. The existence of renal neuralgia cannot be denied. It occurs chiefly in neurasthenia and is said to be evoked by the use of certain irritant drugs, such as cantharides. According to Raynaud and Albarran a visceral crisis of locomotor ataxia may be seated in the kidney, and be manifested by neuralgic pain. Malaria, gout, hysteria, and certain forms of nephritis have also been accused of causing renal neuralgia. The attacks are exactly similar to those of true renal colic and may be accompanied by hematuria, so that they may be very puzzling. The pain is, however, not aggravated by motion, as in stone.

A number of other abdominal conditions, such as cholelithiasis, ulcer of the stomach, aneurism of the aorta, appendicitis, and the like, have been confounded with renal calculi.

When we feel reasonably sure that we have a renal calculus to deal with and also that it is on a certain side, it remains for us to prove, as far as possible, that we are right by all the diagnostic means at our command and also to ascertain if there is another kidney present, and, if so, its condition and working power.

It is said that palpation will in certain cases reveal the presence of stones if they are of a sufficient size. This may be true, but I believe it more probable that the enlarged kidney itself is felt, and that stones could not be detected through the hard walls and cushions of urine and pus.

We can, however, often determine the presence of tenderness and mobility by palpation, as well as the size of the organ, which are important points, inasmuch as an inflamed kidney, especially if it contains a foreign body, is very apt to be enlarged and tender and firmly attached by inflammatory adhesions. A normal kidney cannot, as a rule, be felt, and it is not tender to the touch.

The best method of palpating a kidney is by placing the tips of the fingers of the hand between the last rib and the external border of the sacro-lumbar muscle and to press upward and inward while making counter-pressure with the other hand over the front of the abdomen.

The phonendoscope combined with percussion over the kidney, is said to be of value in delineating the outlines of the organ. It is not, however, absolutely trustworthy as regards the presence of calculi, which it is supposed to indicate by a change in the percussion note.

If we are confident that the kidney on one side is diseased and do not know the condition of the other, we can feel reasonably sure that it is in good working order if it is not enlarged or tender, and if the twenty-four hours urine is of sufficient quantity and contains enough urea. In order to be more certain of its condition we can insert the Harris segregator and examine the urine from either kidney, so as to determine the condition of each. I have used this instrument a number of times and have never had it fail but once, although I can understand how it might give wrong results if not accurately placed after its introduction.

There is, however, an instrument that never fails to tell us which is the healthy or healthier one of the two kidneys, and that is the catheterising cystoscope, provided that one is able to use it; for by this means we can obtain with certainty the urine from each kidney separately without fear of its leaking over the barrier. Unfortunately, however, but few of us are able to use this instrument with any degree of certainty, and I have seen some of the most skilful urologists fail to introduce the catheter, and that, too, in a female patient. Occasionally ureteral catheterisation in the hands of the ordinary practitioner is successful, but more often it is not. I feel, however, that a modern surgeon should never perform a nephrectomy unless he has first catheterised (or had someone catheterise for him) both ureters, has found both kidneys to be present, and the "second kidney" to be healthy enough to eliminate the necessary quantity of urine and waste products after the removal of the diseased organ.

Most of us, however, are not only unable to catheterise the ureter with any degree of confidence in the result, but often cannot even see both of the urethral orifices with an examining cystoscope. I remember a short time ago that together with two of New York's best genito-urinary surgeons, I examined a woman with a large "pus kidney" and not one of us could see the mouth of the ureter.

If the surgeon cannot decide by a visual examination of the bladder that a second kidney is present, and if he does not feel that he can rely upon the Harris instrument, then an exploratory laparotomy is justifiable, as by this means he can at least be sure that a second kidney exists, and can also tell something of its size and general condition. Of course, it may be said that it would be better to go into the loin on both sides and examine the kidneys; that such double incisions would be equally efficacious and would obviate the danger of opening into the peritoneal cavity—all of which is true—but, on the other hand, it would expose the kidney on which the patient must rely principally after an operation to an extra amount of manipulation. Only a few months ago I resorted to this procedure in the case of a woman who was running an intermittent temperature, associated with pain in the right side, in whom the bladder was so small and hemorrhagic that I could not see the ureter.

The clinical history and an off-hand examination does not always tell us whether a patient has nephrolithiasis, and if so, which is the affected side; neither does catheterising the ureters. I will mention, for instance, a case that was still perplexing after ureteral catheterisation.

Merchant, age 48; thin and neurasthenic. Fifteen years ago he had an attack of nephritic colic on the left side. Fourteen years ago he had articular rheumatism and a severe urethral discharge. Twelve years ago he had a colic in his right kidney, lasting eight days. This was the first and last time that he had ever had any pain on that side. Ten years ago he passed some gravel, after which he was well for a few years. For six years he had suffered from dyspepsia, indigestion, headache, dry tongue, flatulence and pain in the back, especially in the left scapular region and loin. He urinated three or four times a day, sometimes oftener.

On examination, two years ago, he had tenderness on palpation in the left kidney. His epididymes on both sides were indurated, especially on the left side. Urethra showed a stricture, 18 French. Urine was alkaline, ammoniacal; specific gravity, 1017; cloudy, abundant sediment; urea, 1.8 per cent.

Some blood, pus, triple phosphates, vesical and pelvic epithelium; no casts. At a later examination, a phosphatic cast of one of the calyces of the kidney was seen. No vesical calculus was found.

The patient left me, promising to return shortly, but did not do so until two years later, *i.e.*, four months ago. His symptoms had remained the same, and he still had pain on the left side, the right being free from pain. His physician had told him that he had a stone in his left kidney. His urine taken from both kidneys by catheterisation showed from the right kidney only one-sixth as much urine as from the left. The urine of the right kidney was darker, of a higher specific gravity and contained red blood cells, pus in masses, renal epithelium, granular, epithelial, and mixed casts and no crystals. The left kidney had secreted six times as much urine, but the latter was of a lower specific gravity and contained but a few red corpuscles and renal epithelial cells. A bacteriological examination of his urine showed no tubercle bacilli, but many gonococci. The result of this examination made with the urine of the diseased kidney, as well as his symptoms and general condition at that time, compared with those of two years ago, showed that the general condition was about the same, also his cystitis, but that his kidneys were in a worse condition; that he had developed a pyelonephritis on the right side and a pyelitis on the left; that possibly the condition of his right kidney might be a pyelonephritis due to ascending infection from the bladder, following his gonorrheal infection of fourteen years before, and that possibly he might have a stone in the left kidney which had always been the seat of his pain.

Accordingly, I advised him to have an *x*-ray picture taken to see what it would show, and in any case to have an operation performed on the right side to explore the kidney, and perhaps to remove it. He failed to do this, however, and sailed to France a few days later, promising to come back for an operation. He went to Contrexéville to take the waters, as they had benefited him on a former occasion. Since then he had evidently changed his mind; for recently I heard that he had been operated upon in Paris, and that the surgeon had removed a large stone from the right kidney.

This particular case is very interesting, as it shows how large a number of gonococci can be found in the urine fourteen years after an attack of gonorrhea; that although his right kidney had only pained him once and then had remained quiescent; but that, on the other hand, although he had been suffering almost constantly pain of every description in his left kidney for fifteen years, the right kidney was really the more seriously affected of the two, and therefore that all these years he had been suffering from renorenal reflex pain, unless he had also a free and movable stone in the pelvis of his left kidney. But above all, this case shows that we must resort to every means of diagnosis in cases of suspected stone, and, had I insisted upon an *x*-ray picture, I would not have made such a mistake.

The Röntgen rays have been employed in the diagnosis of calculi with a fair measure of success. Although Morris thinks (Vol. II., p. 91, *loc. cit.*) that "hitherto they have afforded but little reliable help in this direction," Guyon (*Comptes Rendus de l'Académie des Sciences*, April 21, 1896,) in communicating the experiments of Chappin and Chaumont to the Academy of Sciences in Paris, showed that uric acid is traversed by *x*-rays, but that in spite of this, uric acid calculi may be radiographed in the kidney. MacIntyre, of Glasgow, (*Lancet*, July 11, 1896,) Morton (*Lancet*, June 4, 1898,) and Swain (*Bristol Med. Chir. Soc.*, 1897,) succeeded in determining the presence of renal stones in living bodies, while Sabrazen, Rivière and Garmaud (*Soc. Anat. et Physiol. de Bordeaux*, Dec. 13, 1897,) and Laurie and Leon (*Lancet*, 1897, I., 169) made satisfactory observations upon cadavers. On the other hand, Guyon and Albarran (quoted by the latter, *loc. cit.*, p. 893,) failed to get radiographs of uric acid calculi in living bodies. Lester Leonard (*Phila. Med. Jour.*, 1898, p. 388,) diagnosticated the presence of uric acid calculi in three cases by the use of specially constructed tubes. Leonard's radiographs are of the highest grade, and in his hands the *x*-ray proved of the greatest value in the diagnosis of nephrolithiasis.

The exploratory incision is the "dernier ressort," settling the question of whether a stone is present in the kidney or not. It would seem that this would at once establish the diagnosis, but I will show by the history of a case that I will report further on that this may fail, even when one considers himself fairly careful in his exploration.

*Operative Treatment.*—In the operative treatment of nephrolithiasis, the surgeon should never decide beforehand positively what operation he is to perform, but should be governed by what he has learned of the working-power of the affected and the opposite kidney, by the examination of the urine, the general condition of the patient before and during the operation, and the condition in which he finds the kidney after exposing and inspecting it.

If the kidney is a healthy one, that is to say, if there is no pus in the urine, and if we find no evidence of suppuration in the parenchyma or the pelvis during the operation, then a simple lumbar nephrotomy should be performed. Lumbar nephrotomy consists in making an incision in the loin, exposing the kidney, cutting through its convexity, removing the calculus and then sewing up the kidney. A gauze drain should then be passed down to the organ and the wound in the loin closed, excepting at the point where the drain comes out. This is the ideal operation, and the mortality almost *nil*. But, unfortunately, we find but few such aseptic cases, and those that we have to deal with are usually more advanced. It may be well to say, however, that recovery from the operation is not always assured and I well remember one case which I considered most promising in which the patient died very quickly. This case was operated upon sometime ago, and perhaps I attempted to do too much, as I very carefully closed the external wound after sewing up the kidney, when I saw that the bleeding had ceased. The case had been found to be an aseptic one, and after removing the stone it seemed as if closing the external wound would do no harm. The case was briefly as follows:

B., 19 years old, tall, heavy, with rather a white, pasty face; complained of pains in his stomach, especially marked after exercise, such as playing ball. On these occasions the pain was quite sharp, and sometimes lasted for several hours, and occasionally he noticed blood in the urine. This pain was generally in the left loin, and often caused him to vomit. He urinated when up and about, if active, 7 or 8 times a day, but not at night.

*Examination.*—Tenderness on palpation in the left kidney. Urine from left kidney, pale with reddish-brown sediment; trace of albumin; blood isolated and *en masse;* uric acid crystals, renal epithelium; casts—hyaline, granular, epithelium, blood and mixed. From right kidney, color, amber; slight trace of albumin; few hyaline casts; few blood cells; few uric acid crystals.

*Operation.*—Nephrotomy performed on left kidney. Uric acid stone, freely movable and wedge-shaped, as large as the end of the thumb, found in the pelvis of the kidney. Calculus removed and wound closed. At the end of 24 hours he had passed 24 ounces of bloody urine. Temperature, 102°; pulse, 140; respiration, 36; was vomiting a greenish fluid. His temperature, pulse and respiration continued to go up. On the following day, temperature, 104°; pulse, 180; respiration, 39. Forty-eight hours after the operation the patient's temperature was 102°; pulse, 160, respiration, 48. He had vomited occasionally a yellowish and later a brownish fluid. His bowels had moved, and he had passed 28 ounces of urine. The wound in the abdominal wall had been reopened and drained. There was a slight sero-sanguineous discharge. His temperature and pulse began to go up again, and a few hours later it was 106.4°. The pulse could not be counted, the respiration was 46. Shortly afterwards he died—60 hours after the operation.

Every possible restorative, stimulant, diuretic and palliative had been given, but none seemed to check his rapid decline. No autopsy was obtained.

*Nephrostomy.*—If pus be found at the operation, after the kidney is opened and the stone removed, a drainage-tube should be inserted into the pelvis of the kidney and the organ sutured on either side of it. Gauze is then packed about the tube down to the kidney, and the abdominal wound is closed as already mentioned. This is usually a successful operation if we succeed in finding the stone, but sometimes the stone is not found. A case in point was the following:

Housewife, age 36. For about eight years she had had pain in the right lumbar region. She had attacks of fever which would last for a few hours to a few days, which had been called malaria by several physicians. The right side for some time seemed fuller than the left. The woman had given birth ten days before to a seven months' fetus, after which the tumor was noticed, which, it was thought, had caused pressure against the uterus and had given rise to a miscarriage.

Upon examination the woman appeared thin and sallow-looking. Temperature, 101°; pulse, 94; respiration, 36. Tumor was evident in the right lumbar region extending from the ribs into the iliac fossa and in front beyond the median line. It seemed to be a case of movable kidney with renal obstruction due probably to a kink in the ureter. Patient was sent to the hospital for operation and four hours later her condition became critical. Temperature, 104.6°; pulse, 140; respiration, 46. No defined tumor could now be felt; a large mass, however, occupied the entire right side of the abdomen; the drive in the ambulance had evidently caused a rupture of the capsule of the kidney and a leakage into the post-renal space. Patient had a vaginal discharge with some fetor. Urine—color, dark; specific gravity, 1022; albumin, pus, blood and urates. The patient's condition was critical and she was kept on opiates and stimulants. On the following day, temperature 99.4°; pulse, 86.

Then I determined to make a rapid exploratory incision, consequently a lumbar incision was made down to the kidney and about a quart of pus was evacuated. A rupture was found in the lower pole of the organ; an incision was now made into the pelvis of the kidney, the calyces were palpated and the ureters were catheterised, but no stone was found. After this operation she still ran a temperature for several days, when she was curetted and an abscess was found in the broad ligament, which was opened and drained. She still ran a temperature; there was a considerable drainage of pus from the kidney, and a nephrectomy was performed. The kidney was found to be a scirrhous mass and a stone one and a half inches in diameter was found in a pocket on the other side of the organ communicating with the cavity of the pelvis by a circular route. Her temperature and pulse dropped immediately after the operation, but her puerperal septic condition finally caused her death.

This is an exceedingly interesting case, for lumbar incision was performed, the kidney opened from the rupture into the pelvis in its lower pole. The pelvis was palpated and probed without finding the stone, and yet after its removal, by bending a probe in a certain direction, it could be passed from the point of rupture into the pocket where the calculus was encysted.

*Nephrectomy.*—If the parenchyma is involved and abscesses are present, or if the kidney is but a sclerosed mass of tissue, or nothing more than a suppurating sac containing stones, then a nephrectomy may be performed instead of a nephrotomy and the organ removed.

There are arguments for and against both these procedures, but the condition of the individual is the main factor to be considered, and the patient's life must never be sacrificed for the sake of performing one operation that is considered more radical than another. A case showing an unfortunate coincidence of disease in the other kidney which had not been recognised before operating, was as follows:

Housewife, aged 49; has been suffering for about two and one-half years with pain and a feeling of distress in umbilical and right lumbar regions, which was diagnosticated as dyspepsia, and for which she had been treated for some time without relief. About six months ago, noticed a small tumor in right lumbar region, which was tender and painful. At times the pain subsided, and at other times it was exaggerated; however, she became much worse with frequent vomiting, loss of appetite, constipation; the tumor grew slowly; the patient was weak and emaciated. Temperature slightly elevated at night. Urine: color, light; specific gravity, 1016; slight amount of albumin; no casts; some pus.

The tumor extended from the ribs on the right side to the iliac region, and as far as the median line. It was hard and tense. Four days after admission, the urine contained a great amount of pus, but later in the day became clear again. This happened quite frequently, but usually the urine was clear and of a low specific gravity. The right ureter was evidently completely occluded at times, during which period the left kidney secreted comparatively normal urine, showing it to be in a healthy condition.

A lumbar incision was made, and a large kidney was brought to view, the capsule of which was of a blue-gray color. There was no perirenal fat. The kidney was very easily freed, and the vessels of the pedicle were ligated, the organ removed, and then the ureter was tied off and severed.

The kidney was a very large one, and upon opening it, there was found about a pint of pus and a number of phosphatic calculi, from the size of a hickory nut to that of an English walnut. The parenchyma was entirely atrophied.

The patient passed no urine for three days when she died of uremia. The condition of the other kidney was that of acute congestion and granular parenchymatous degeneration. It was thought that the other kidney had been comparatively healthy at the time of the operation. This sudden death seems to be rather remarkable, when I consider that on one occasion I was so unfortunate as to remove the only kidney that the patient possessed, and yet he lived for eight days and was very comfortable during six.

I remember a number of cases of secondary nephrectomy some of which were performed by me, the patients having been referred to me for this operation; one in which a nephrotomy had been performed, but the condition of the patient was not sufficiently good at the time of the operation for the removal of the organ, and it was thought best to drain and wait until the patient's health had been built up, at the same time watching his general condition and that of the urine; and yet another in which a renal fistula remained, and the amount of suppuration was such that it seemed to weaken the patient and was pulling him down instead of allowing him to improve.

Secondary nephrectomy is a very difficult operation, as in such cases the kidney has lost its form, the adhesions are often more dense than at the preceding operation and there is pus and urine leaking into the operative field,—a great source of danger, as the peritoneal cavity is at times opened in dissecting away dense peritoneal adhesions from the kidney capsule. In such cases, it is better at times not to do too much, as is illustrated by the following case:

A married woman, 28 years of age, who had been operated upon by nephrostomy and had left the hospital with a renal fistula, came back about six months afterward for further treatment, saying that she was unfit for her household duties on account of her ill health, due to this suppurative condition. I made an incision around the fistula, went down to the kidney, removed the tissue comprising the fistulous tract and began to enucleate the kidney. The kidney seemed to be freed very easily for a case of secondary nephrectomy, and the mass in my hand seemed to be large enough to be a kidney of good size, although it was not so hard and sclerosed as usual. On closer inspection I found I had shelled the kidney away from its capsule which was very thick, and firmly adherent to the surrounding tissues. I could easily have passed a ligature about the mass, ligated it and then either removed it (decortication), or allowed it to slough away, but instead I removed the kidney capsule with a great deal of difficulty, having to break up adhesions to the surrounding viscera, the result of which was, that the patient died in a few hours from shock. A case of this kind shows us that, although the result would have been most satisfactory had the nephrectomy been successful, it was, instead, the direct means of shortening the woman's life.

Cases of this kind teach us to proceed with more care, and not to resort to measures that would occur to us when we felt bolder. Shortly after this last case, a most unusual case came under my care, which shows us what can become of a kidney in time, and which might have resulted as disastrously as the last case, had I pursued the same tactics.

A musician, aged 48, had been suffering for sometime with pain in the left side. Two years ago an abscess developed in the left lumbar region. This was opened, and since then there has been a constant discharge from the fistula, sometimes more profuse than at others. This discharge began growing less profuse of late, but it was still a great cause for discomfort, and he had made up his mind on his visit to New York to have an operation performed. An examination of the fistula showed that it extended down in the direction of the kidney. The urine was normal, with the exception of some pus and a few vesical and pelvic epithelia.

An incision was made over the sinus and along the outerside of the erector spinæ muscle from the 12th rib downward, with the idea of discovering the source of the abscess. This led down to a space beneath the deep muscular layer where a foreign body was found which proved to be a phosphatic calculus an inch long, and half an inch thick. Continuing my dissection, I found a thin sclerosed kidney, the parenchyma of which was almost entirely destroyed, possessing simply a very much thickened capsule bound down by dense adhesions that added to its thickness. It was evidently the remains of a calculous pyonephrosis that had broken into the perinephritic tissue, allowing the stone to escape, thus giving rise to a perinephritic lumbar abscess. This was opened by a small incision, evacuating the pus and leaving a renal fistula. The stone was lodged between the posterior wall of the kidney and the muscular layer of the abdominal wall adjoining it. The kidney could not be freed without risk from its anterior adhesions, and so the posterior half was removed and the anterior half was allowed to remain. The patient made an uneventful recovery and left the hospital with the incision nearly healed.

The case is instructive as showing how a stone can remain outside the kidney for presumably a long time and how a kidney can become so changed that it is not recognized at first as such.

I will mention still another interesting case of secondary nephrectomy to illustrate some complications which may take place:

A laborer, aged 22, entered the hospital saying that eleven days before he had fallen from a height of twenty-five feet, striking on his left side. This hurt him so much that he could not get up, so some of his friends carried him away from the spot, and after a short rest he walked home. Before this he had always been well, but a few days after the accident he noticed a painful swelling on the left side. On entering the hospital, there was a tumor occupying almost the whole abdominal cavity, although the left loin bulged more than the right. The urine was negative, the temperature 101.6°, the pulse 88, and the respiration 20.

An exploratory incision was made over the tumor in the left semilunar line, and the peritoneum was cut through. There was practically no peritoneal cavity, for the back part, instead of being a hollow in which the viscera lie, was pushed up against the anterior wall like a dome, and the intestines were stretched out like so many ribbons. The tumor was seen to be postperitoneal, the anterior incision was accordingly sewn up, and a posterior, lumbar incision was made on the same side into the retroperitoneal space, when 2½ gallons of a wine-colored fluid, consisting of mixed blood and urine, escaped. There was a rent in the lower part of the kidney, evidently caused by the fall. The pelvis was opened through the rent, but no stone could be felt. A tube was put into the kidney for drainage. The patient was much relieved by the operation, but began to swell again at the end of a week. It was found that the tube had slipped between the erector spinæ muscle and the skin. Another opening was then made down to the kidney, 3½ quarts of urine and pus evacuated, and the tube reinserted into the kidney. The kidney was a large sac, while the bladder urine was normal, showing that there must have been an impediment in the ureter.

The patient was again very much relieved, but continued to run a slight temperature. As he was losing ground, I decided to perform a more radical operation. On reopening the incision, I found that the kidney consisted of a large sac, the parenchyma having been destroyed; that the mouth of the ureter and its first portion was very much dilated, and that the adhesions were very dense about the kidney, extending down to the ureter.

The duct was very much thickened as far as could be reached, and just below the brim of the pelvis there was probably a calculus, with ureteritis and a mass of adhesions surrounding it. A ureteral catheter could not be passed beyond this point. It seemed to me that this kidney was useless, and that it would be easier to perform nephrectomy than to remove the stone from the ureter and that nephrectomy would be the better procedure in the patient's condition. The mass of adhesions were seized above and below with hysterectomy forceps, transfixed and tied. The pedicle was very wide, spread out like a fan, and the vessels were adherent. It was accordingly transfixed, and when one of the vessels was wounded, giving rise to considerable hemorrhage, a double ligature was tied upon it. As the bleeding continued in spite of this, a curved hysterectomy clamp was placed upon the pedicle inside of the ligature. The patient was then put to bed with the forceps in place, and the latter were removed in four days.

This is the second time that I had to leave forceps in place on a renal pedicle. It was very trying for the patient, who was obliged to lie on his side for four days, for if he turned over on his back, the ends of the forceps might puncture the intestines, or the blades might spring, giving rise to secondary hemorrhage.

*Position of the Patient.*—There are many different positions in which the patient may be placed in kidney operations. Personally, I prefer having them lie at full length on the unaffected side, with a sand-bag of sufficient size under the loin to cause the affected side to bulge to its fullest extent, thus giving the greatest possible space between the ribs and the iliac crest. A sand-bag of similar size is to be placed under the patient's neck, on the same side.

The *incision* that I usually make is a semilunar one, extending from just above the angle made by the erector spinæ with the last rib, along the outer border of the erector spinæ, nearly down to the crest of the ilium, where it curves forward to the anterior superior spine. Naturally, I do not always cut so far, but this is the line of incision.

In *palpating* the kidney after it has been exposed, the organ is taken between the thumb and fingers of the one hand, while with the thumb and fingers of the other hand, it is gone over its entire extent, pressing into the calyces on either side, and following the dilatations down the ureter.

*Needling* of the kidney is of little value, and I do not know that I have ever accomplished much by this means.

*Aspiration* is, on the other hand, very useful if the kidney is pyonephrotic, hydronephrotic, or sacculated, as it allows us to puncture it and then, by attaching a piece of tubing to the canula, to empty the contents outside of the operative field. The cavity can also be washed out through this instrument. After the kidney cavity has been emptied, stones can be more easily felt through the walls than when it is full of fluid so that the stones have a fluid cushion over them.

In *nephrotomy* the incision is the same as just described, the kidney is opened through its convexity, and then closed by suture of catgut going through the cortex at the superficial part of the cut. I use alternating sutures of chromicized, and plain catgut, and put in a gauze drain down to the surface of the kidney.

In *nephrostomy* the operation is the same except that a tube is inserted into the pelvis of the kidney and tied in for some days. I frequently leave a drain in for two weeks, washing the pelvis out twice a day with a boric acid solution, and every other day with a solution of nitrate of silver. Some prefer inserting a ureteral catheter up into the pelvis of the kidney through the bladder. Others prefer passing it down through the pelvis into the bladder, and then catching the end with a lithotrite and pulling it out through the urethra. If that is done, it is well to attach a thread to the kidney end of the catheter to prevent it from being drawn into the bladder.

*Nephrectomy* is a much more dangerous operation than nephrotomy, as there are more adhesions to be broken up, and there is therefore more danger of breaking into the peritoneal cavity, the colon or duodenum. In the first instance, a fatal peritonitis may ensue, while in the other case an intestinal fistula would result. I have cut through the peritoneum accidentally in two different cases. In the first instance I cut through both the peritoneum and the kidney in separating a sacculated organ; but, notwithstanding that there was some pus and urine in the cavity, by quickly seizing the sides of the rent, sponging it, and sewing it up, no infection resulted. In another case I tore the peritoneum over the liver, so that I could see that organ but I walled it off and drained it effectively, so that there was no infection of the cavity. Another reason why nephrectomy is more dangerous is because so much extra work is suddenly thrown upon the other kidney, which has perhaps been incapacitated somewhat by the anesthetic.

*Treatment of the Pedicle.*—The pedicle varies in different kidneys. Sometimes the ureter can be separated with ease from the rest of the pedicle and the ligature passed between the vessels, while at other times they are all three *en masse*. The ureter can usually be separated, and after clamping the pedicle, a blunt-pointed needle, carrying a ligature, can be passed through it and the ligature tied in a double loop on the side. Later, if necessary, another ligature may be applied over the entire pedicle. Occasionally, the ligature does not hold well, but slips, and then there is a bad hemorrhage. In such cases clamps must be immediately put on the pedicle, and the hemorrhage arrested.

In two cases operated upon by me, clamps had to be used and left *in situ* for four days. In one of these a vessel of the pedicle was transfixed, and in the other the ligature had slipped. The safest material for ligatures is braided silk. In order to be on the safe side, if everything fails, I have devised a clamp which will fit any pedicle and may be left *in situ* without danger of injury to the intestines by pressure.

### PROGNOSIS.

The prognosis of operations for renal calculi is always serious, when we remember the risk of anuria and of septic complications, and also the effects of nephrolithiasis upon the parenchyma of the kidneys. The results of operations collected by Albarran, Gross, Legueu, McCosh, Morris and Newman, show that early operation is advisable and that nephrotomy is the operation of choice, giving the smallest mortality. Legueu gave a mortality of 5 per cent. in his series of nephrotomies and Morris still reduced this figure to 2 per cent. A long-standing or permanent fistula occurred in his cases in only 6 per cent. after nephrolithotomy, nephrotomy, and nephrectomy for stone.

*Results of Operations.*—In a collection of statistics made by Morris from the operations published in America and England between 1883 and 1893 there were: (1) 115 nephrotomies with 103 recoveries and 12 deaths—13.8 per cent.; (2) 32 nephrectomies with 25 recoveries and 7 deaths—21.8 per cent.

The results vary greatly according to the presence or absence of septic complications in the kidney, thus: (1) In aseptic cases the mortality after nephrostomy was less than 4 per cent.; (2) in septic cases the mortality after nephrostomy was 20 per cent, but has been as low as 10 per cent.; (3) in septic cases (*a*) primary nephrectomy gave mortality of 33 per cent., which of late has gone down to 20 per cent.; (*b*) secondary nephrectomy gave 23 per cent. mortality.

### AFTER-TREATMENT.

The after-treatment of patients who have passed through an operation for stone in the kidney is very important, as upon this may depend the ultimate result of the operation.

The best position for such a patient is on the back and with pillows arranged in such a way that there is no pressure upon the wound. This detail is especially important in cases where drainage is used.

The dressing of such wounds does not differ from ordinary operative wounds of the abdominal cavity. In septic cases drainage should be maintained according to the indications, until the fistula heals up. For this purpose, dry gauze wicks will be usually found effective, but sometimes drainage-tubes placed in the pelvis of the kidney are necessary. In ordinary aseptic nephrotomies, the gauze should be inserted down to, and not into, the kidney. A binder is placed over the gauze pad in the loin.

The immediate after-treatment concerns itself with moderating the shock and does not present any special peculiarities. The danger of shock being over, that of anuria is to be foreseen and prevented as much as possible by all the means at our command. These include the administration of bland fluids; at first hot water, then milk and alkaline mineral waters, saline injections and infusions; hot packs; diuretics; diaphoretics; purgatives, and the use of pilocarpine subcutaneously. Retention of urine should be prevented by passing the catheter as often as is necessary to be sure that the bladder is empty.

The bowels should be moved by salines on the second day after the operation, aided perhaps by enemas of soap-suds. The dressing may be left undisturbed until the third day if the temperature remains normal, and if there is no excessive discharge from the wound. Otherwise it should be renewed sooner or more often according to the amount of discharge and general condition of the patient. Perineal suppuration and the formation of pockets of pus may take place, requiring opening and draining until healed.

The duration of the after-treatment is usually two weeks, but this period is often prolonged to four or six weeks by suppurative complications. When the wound has finally closed, an abdominal belt or some other means of support should be worn to diminish the danger of hernia, particularly in cases in which an extensive abdominal lumbar incision has been made.

75 West Fifty-fifth Street.

